# Autolysis and Cell Death Is Affected by pH in *L. reuteri* DSM 20016 Cells

**DOI:** 10.3390/foods10051026

**Published:** 2021-05-09

**Authors:** Tahl Zimmerman, Salam A. Ibrahim

**Affiliations:** Food Microbiology and Biotechnology Laboratory, Food and Nutritional Sciences Program, 163 Carver Hall, North Carolina A&T State University, 1601 Market St., Greensboro, NC 27411, USA; ibrah001@ncat.edu

**Keywords:** LAB, autolysis, cell preservation, almond drink

## Abstract

A key obstacle to the successful delivery of a probiotic to the consumer is maintaining viability of the live cells during storage, a challenge for the beneficial *Lactibacillus reuteri*. Three processes play a role in the reduction of viability: autolysis, cell death, and cell weakening. Using a phosphate induction model of autolysis, the initial aim of this project was to discover novel molecular determinants of autolysis in *L. reuteri*, with the long -term goal of elucidating new strategies for increasing viability. We employed a 2D Native/SDS-Page method to monitor changes in protein expression over time; however, the result was that excess phosphate did not induce noticeable changes in expression patterns. On the other hand, we found that pH affects both the rate of total viability and autolysis, as seen with other species of LAB. In addition, we found that the phosphate model of autolysis may not be sufficient to explain how autolysis is triggered in *L. reuteri*. Two parameters appear to modulate the pH in media containing *L. reuteri* cells: overall buffering capacity and the presence of a carbon source. Ultimately, phosphate sources appear to facilitate autolysis by maintaining pH in the media via a higher buffering capacity. In addition, the alkaline sugar free almond drink appears to be a promising possible preservative for *L. reuteri*.

## 1. Introduction

Probiotics are defined by the WHO as “live microorganisms which when administered in adequate amounts confer a health benefit on the host” [1]. For example, *Limosilactobacillus reuteri* (formerly known as *Lactobacillus reuteri* [2]) has been found to lower cholesterol [3], to have anti-inflammatory effects [4] and to block carcinogenesis [5]. Consuming *L. reuteri* as a true probiotic would thus be beneficial to human health. However, one key obstacle in the delivery of *L. reuteri* is the challenge associated with preserving viable cells during storage. Only viable cells are capable of attaching to the gastrointestinal system, and large numbers of viable cells are necessary in order for a true probiotic to deliver its health-benefits [6]. However, cell viability is limited by the processes of autolysis, cell death and cell weakening. Autolysis is a process whereby cells provoke their own lysis in response to a chemical insult or physical injury [7]. Autolysis is mediated by enzymes known as autolysins, which bind lipoteichoic acid on the cell surface before cleaving the peptidoglycan layer, leading to destabilization of the cell wall and lysis [7]. The process of autolysis in lactic acid bacteria (LAB) is affected by many factors such as pH [8], temperature [9], and ionic strength [10]. Likewise, autolysin enzyme activity in LAB is influenced by pH [11].

The internal (cytoplasmic) signaling pathways that trigger autolysis are not well articulated [7]. A clear understanding of the molecular factors that modulate autolysis and cell viability can be useful for developing new strategies to preserve viability as the maintenance of cell viability is crucial for the long-term storage of *L. reuteri*.

In a prior study, we utilized an already established phosphate induced autolytic system [12] to screen conditions for blocking autolysis and discovered that choline and its analog Hemicholinium-3 (HC-3) could be used to prevent autolysis [13] in the phosphate induced model. In addition, we observed that that phosphate treatment led expression differences with respect an untreated control in a 1D SDS-PAGE [13]. The aim of the current study was to further understand the expression changes induced by phosphate, in order to identify novel molecular determinants of autolysis.

In order to gain better resolution of the protein expression patterns we employed a 2D Native/SDS-PAGE system to monitor changes in cytoplasmic protein expression over time in the presence and absence of excess phosphate. The 2D Native/SDS-PAGE system is a well-established method for studying proteins in their native form [14,15,16] and was chosen in order to capture changes in expression as well as possible post-translational and conformational changes, and changes in the oligomeric states of proteins.

## 2. Methods and Materials

### 2.1. Induction of Autolysis

A phosphate-induced autolytic model [12] was used to assess expression changes in *L. reuteri* strain DSM 20016 (BioGaia, Stockholm, Sweden). Cells were cultured at 37 °C in Man, Rogosa and Sharpe media (MRS) for 12–13 h until an optical density at 600 nm (OD_600_) of 2.0 and a cell density of ˜9 log CFU/g were reached. The cells were centrifuged at 1000× *g*, washed 3× with fresh MRS, and resuspended with either fresh MRS alone or fresh MRS containing 6% potassium phosphate (Fisher Scientific, Waltham, MA, USA) also known as MRS-KP, to a final volume equal to the original culturing volume of 500 mL. Cell resuspensions were then aliquoted at 50 mL and incubated at 37 °C.

Fifty mL samples were removed daily in order to obtain CFU counts, OD_600_ readings and 2D Native/SDS-Page analysis. CFU counts were determined by plating samples on MRS-agar plates followed by incubation for 24 h at 37 °C.

### 2.2. D Native/SDS Page Analysis

Fifty mL of MRS and MRS-KP treated cells were washed 3× with 62.5 mM Tris pH 6.8. The supernatants were discarded, and the pellets were stored at −20 °C. The cells were then resuspended with 1 mL of 62.5 mM Tris pH 6.8 (Buffer 1) and lysed by sonication. Sonication is a well -established method for releasing cytoplasmic proteins from cells [12]. Thirty μg of each sample were loaded onto precast native gels (Mini-Protean TGX, Biorad, Hercules, CA) after 20 μL of 5× loading buffer was added and sample volumes were normalized to 60 μL by adding buffer 1. The gels were run for 210 min at 80 V in a Mini-Protean Cell (Biorad), and then incubated in Buffer 2 (3% Tris base, 14.4% glycine, 1% SDS) for 30 min. Lanes were cut out using a razor, and each lane was then placed over individual 12% SDS-Page gels which were run for 60 min at 200 V. The gels were then stained by Coomassie and analyzed using the Amersham Imager 600 (GE Healthcare).

### 2.3. Monitoring the Effects of pH Adjustment of Consumed Media on Autolysis

Cells were cultured as described above, aliquoted into 50 mL tubes, and harvested by centrifugation. Cell pellets were kept on ice. The MRS supernatants (uMRS) were not discarded, but instead were adjusted to a range of pH values of 4.5 to 8.5 using NaOH, and then filtered with a 0.22 μm filter. Each 50 mL tube containing a cell pellet was then resuspended in 50 mL of MRS supernatant adjusted to a different pH. The tubes were then incubated at 37 °C for a 4-day period. CFUs, ODs and, pH were monitored during this period.

### 2.4. Monitoring the Effects of HC-3 on Autolytic Induction

HC-3 is a known blocker of cell autolysis [13]. The cells were cultured as described above and then either resuspended in uMRS adjusted to pH 6.5, uMRS adjusted to pH 6.5 + 6% phosphate (uMRS-KP), uMRS adjusted to pH 6.5 with 2.7 mM HC-3 (uMRS-HC-3), or uMRS adjusted to pH 6.5 with 6% phosphate and 2.7 mM HC-3). Resuspensions were incubated at 37 °C for a 4-day period. OD_600_ values and CFU counts were taken at zero time and on day 4.

### 2.5. Monitoring the Effects of pH Adjustment of Fresh Media on Autolysis

The cells were cultured as described above, harvested by centrifugation and then washed and resuspended in fresh MRS) or fresh MRS-KP adjusted to a range of pHS (4.5–7.5). Cells were then incubated over a 4-day period at 37 °C.

### 2.6. Monitoring the Effects of Unsweetened Almond Drink on L. reuteri Cells

Cells were cultured as described above, harvested by centrifugation, washed 3× in fresh almond drink and then incubated as described above.

### 2.7. Stastical Analyeses

Statistical analyses were carryout out with the SAS program v 9.4 (SAS Inst., Cary, NC, USA). Significantly different means (*p* ≤ 0.05) between the effects of MRS, MRS-KP, and uMRS, pH, and the addition of HC-3 on the growth (OD600) and viability (log CFU), of *L. reuteri* were separated using Tukey’s Honestly Significant Differences test.

## 3. Results

### 3.1. Adding Phosphate Leads to Autolysis but Not to Observed Changes in Protein Expression

**Figure 1:**
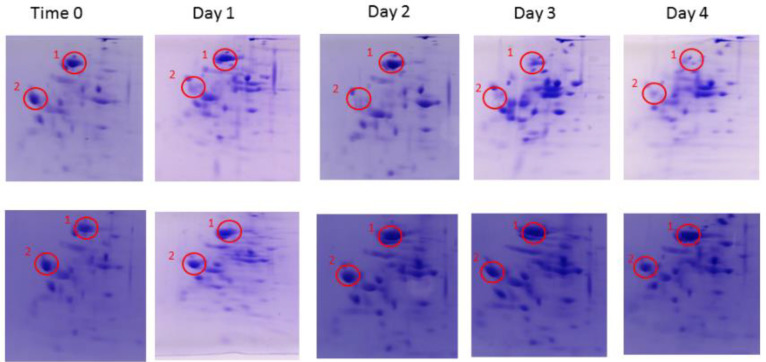
2D NATIVE/SDS-PAGE. A sample was taken from MRS (Top) and MRS-KP (Bottom) incubated cells each day over 4 days and analyzed. There were noticeable changes to the spot pattern over the 4 days in the MRS condition. For example, when observing Spot 1 and Spot 2, a clear reduction in expression was observed. With regard to the MRS-KP condition, the configuration in the 2D NATIVE/SDS-PAGE changed little compared to zero time.

We employed a 2D Native/SDS-Page Gel time lapse model in order to monitor changes in expression of cultured *L. reuteri* cells in response to excess phosphate over a 4-day period. Unexpectedly, we found that in the MRS-KP (MRS + 6% phosphate) condition, no substantial changes in expression patterns were observed with respect to the zero-time control (Day 0, Figure 1, Bottom). In the MRS condition, substantial changes in the expression pattern were observed over the 4-day period, including a drop in expression in a number of species (Figure 1, Top, peak 1, peak 2). This was a surprising and counterintuitive outcome. Our hypothesis was that at zero time, the cells were already primed for autolysis whether they were exposed to excess phosphate or not and that this was the reason that pronounced expression changes were not observed in the presence of excess phosphate. Therefore, the phosphate-induced model alone was not sufficient to explain the observed autolysis in the presence of excess phosphate and, consequently, other factors such as pH must play a role in phosphate induction of the autolytic process of *L. reuteri.* The pH is a known autolytic factor [9]. Importantly, from Day 1 to Day 4 of the MRS condition, the pH value of the media was below 5. An acidic environment can lead to DNA and protein damage in microbes [17], as well as protein unfolding and shifts in protein expression [18]. These phenomenon may help explain why changes were observed in the gels of the MRS condition.

### 3.2. pH Value Is a Factor in Both Autolysis (Phosphate and Non-Phosphate Induced) and Cell Viability in L. reuteri

**Figure 2:**
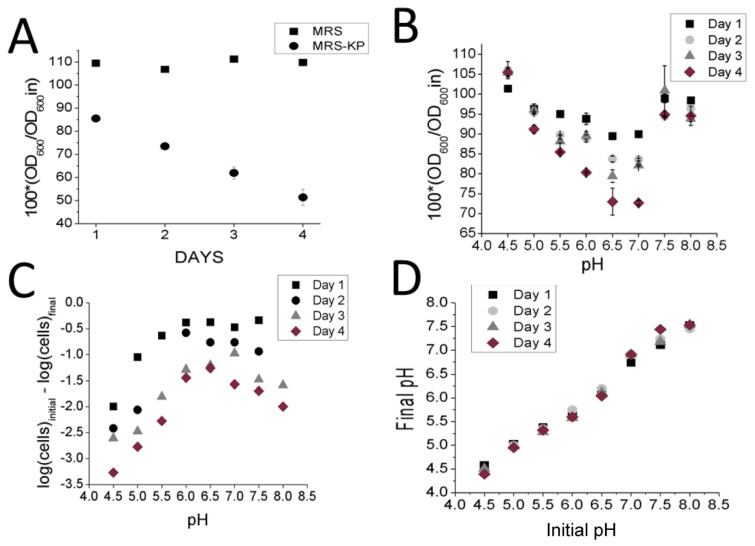
Analysis of pH effects on autolysis and cell viability (**A**) Changes in OD_600_ values observed with induction of autolysis by MRS-KP (starting OD) (**B**) Changes in OD_600_ values observed with induction of autolysis of cells resuspended in uMRS adjusted to different pH values (**C**) Changes in CFU counts observed of cells resuspended in uMRS (starting CFU count) (**D**) Changes in PH of cells resuspended in uMRS adjusted to different pH values.

If the cells were already primed for autolysis prior to being exposed to excess phosphate, why was autolysis not observed in both the MRS and MRS-KP conditions? Our hypothesis was that by lowering the pH in the initial *L. reuteri* cells from the initial pH of 6.5, non-phosphate induced autolysis would be blocked. Extreme pH values are known to block the activity of the autolysin enzymes required to mediate autolysis [7]. Extreme pH values are also known to limit the number of viable *L. reuteri* cells over time in comparison to neutral pH values [19]. In addition, *L. reuteri* is known to produce lactic acid, and excess amounts of lactic acid have been shown to inhibit *L. reuteri* growth. To test this proposition on *L.reuteri*, we cultured cells in MRS, recovered the cells and adjusted the consumed MRS (uMRS, initial pH 4.5) to higher pH values, in the absence of excess phosphate. We then resuspended the cells in uMRS adjusted to different pH values. As expected, autolysis was slow or nonexistent at lower pH values, since at day 4, the change of OD_600_ was minimal with respect to day 1 at a low pH value. (Figure 2B). The highest decrease in OD_600_ was found at pH 6.5 at day 4 (Figure 2B). This decrease in OD_600_ value was found to be statistically significant (*p* < 0.05) with the respect to the decrease observed on day 1. Therefore the most pronounced autolysis was observed at pH 6.5 (Figure 2B). This result is consistent with results showing that the optimal pH for autolysis to occur is pH 7 in *Lactococcus lactis* [10], that the optimal pH is 6.5 for *Lactococcus acidophilus* [8] and that the highest enzymatic activity of AM2 autolysin of *Lactococcus lactis* is between pH 6.0 and 7.5 [11].

An inverse pattern was observed for reduction in cell viability: the least prononunced decrease in cell viability between Day 1 and Day 4 was observed at pH 6.5. The most pronounced difference in viability decreases between Day 1 and Day 4 occurred at pH 4.5, a statistically significant observation (*p* < 0.05) (Figure 2C). Predictably, when uMRS was used, pH values remained steady over time (Figure 2D). Presumably, this was because uMRS no longer contained the nutrients for the *L. reuteri* cells to generate further quantities of lactic acid [20]. This hypothesis is also consistent with data showing that depleting glucose from the media leads to both a higher pH and an increase in autolysis in *L. lactis,* and that excess glucose led to the inverse outcome of low pH and inhibition of autolysis [21]. This result confirmed that pH value played a role in both cell viability and autolysis in *L. reuteri* cells and that these two processes were not necessarily linked. Since excess phosphate was not added in these experiments, this result suggests that *L. reuteri* cells grown to a stationary phase were indeed primed for autolysis in the absence of phosphate, a model consistent with the expression patterns observed in the 2D SDS-Page gels (Figure 1).

### 3.3. pH Induced Autolysis Is Blocked by Hemicholinium-3

**Figure 3:**
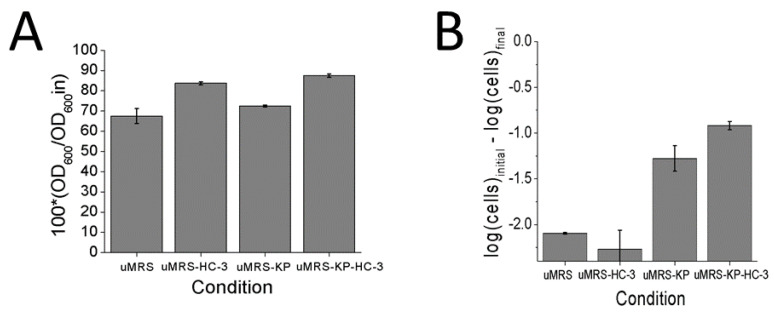
Analysis of the effects of phosphate and HC-3 on cells induced to autolyze by adjusting the pH of consumed media (uMRS) to pH 6.5 (**A**) Changes in OD_600_ values observed between zero time and day 4 (**B**) Changes in CFU counts observed between zero time and day 4.

Hemocholium-3 is an analog of choline that had been previously reported to interfere with the autolytic process in *L. reuteri* cells and minimized the loss of viability [13], presumably by interfering with the interaction between autolysin molecules and the peptidoglycan molecules. In order to confirm that the pH induced autolysis functioned in a similar manner to that of excess phosphate induced autolysis, HC-3 was added to cells in used media (uMRS) or uMRS + phosphate (uMRS-KP) and adjusted to pH 6.5. HC-3 was previously shown to block both phosphate induced autolysis and to minimize losses in cell viability over time [13]. The addition of phosphate did not significantly increase autolysis with respect to the nutrient depleted media uMRS alone (Figure 3A, *p* > 0.05). HC-3 blocked the autolytic process in both the phosphate (*p* < 0.05) and non-phosphate (*p*< 0.05) conditions, with respect to the non-HC-3 controls (Figure 3A). This result would suggest that autolysis in the phosphate and non-phosphate conditions proceeded along the same molecular pathway and that phosphate might not be the only factor triggering the autolytic process in this model.

### 3.4. Phosphate Alone Cannot Induce Autolysis

**Figure 4:**
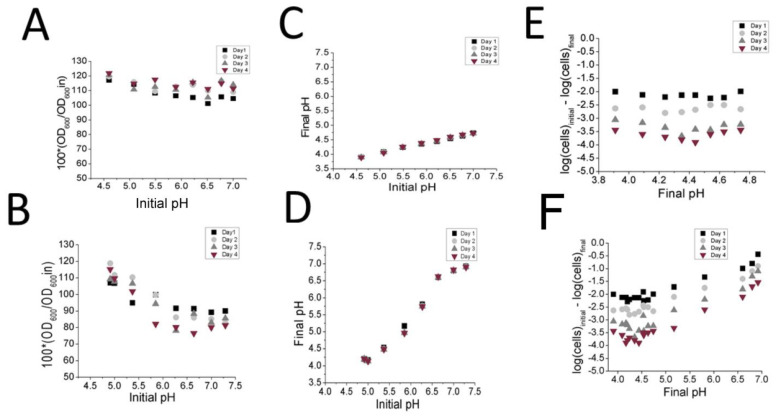
Analysis of pH effects on autolysis and cell viability when resuspending cells in fresh MRS (**A**,**C**,**E**) or fresh MRS-KP (**B**,**D**,**F**) adjusted to various pH values. Changes in OD_600_ (**A**,**B**) pH (**C**,**D**), and CFU counts (**E**,**F**), were monitored over 4 days at 37 °C.

In order to confirm whether or not phosphate alone could induce autolysis, the cells were resuspended in either fresh MRS or fresh MRS-KP media adjusted to a range of pH levels. The result was that no significant decrease in OD_600_ values were observed in any MRS/initial pH condition between days 0 and 4 (Figure 4A) presumably because the pH had dropped to low values on Day 1 (Figure 4C). Consistent with this result, the drop in cell viability between Day 1 and Day 4 did not significantly differ according to initial pH values (Figure 4E). MRS-KP did not induce a significant change in OD_600_ values at lower initial pH values (Figure 4B) between Day 1 and Day 4, while a difference in OD_600_ change was observed at pH 6.5 between Day 1 and Day 4 (*p* < 0.05) pH values did not drop as dramatically with MRS-KP at high initial pH values (Figure 4D), and cell viability varied according to the initial pH values (Figure 4 F). With regard to these results, it appears that the additional 6% phosphate did not induce autolysis per se, but rather facilitated autolysis by maintaining a pH value via an increased buffering capacity. When a high initial pH that favored autolysis was tested (pH 5.5–7.5), the additional phosphate simply maintained that pH, thereby facilitating autolysis. This result was consistent with the much higher buffering capacity measured between pH of 6.5 to 4 for MRS-KP (129.1 ± 0.3 μmoles HCL/pH unit) compared to that of MRS (38.1 ± 0.2 μmoles HCL/pH unit).

### 3.5. Almond Drink Can Both Maintain pH and Slow Losses in Cell Viability

In order to test the proposition that carbohydrate depletion would lead help to preserve *L. reuteri* cells, we used commercially available unsweetened almond drink (UAD) as a model as UAD contains no sources of sugars. In addition, an alkaline pH of 7.5, UAD was found to have a lower buffering capacity (30.4 um μmoles HCL/pH unit) than both MRS and MRS-KP. Therefore, the buffering capacity would play less of a role in cell preservation than it would have done with MRS and MRS-KP. In addition, UAD was already known to be a poor environment for bacterial lactic acid production compared to UAD’s sugar sweetened alternatives [22]. Moreover, lactic acid production ceases when the sugar in the culturing media is consumed [20]. In addition, fermented products made with sweetened almond drink lose substantial viability during storage [13,23,24]. In light of these facts, UAD was thought to be the ideal model for testing the proposition that a low sugar medium would be ideal for maintaining a favorable pH and therefore preserving *L.reuteri* cell viability over time.

**Figure 5:**
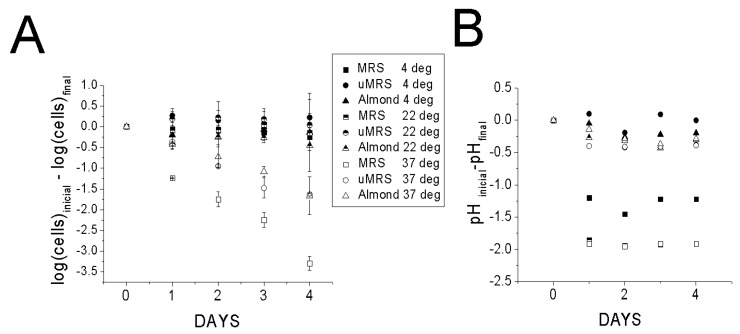
Survivability of *L. reuteri* cells in MRS (pH 6.5), uMRS adjusted to a pH of 6.5, and almond drink (pH 7). Three temperatures were monitored: 4, 22, and 37 °C for each condition. CFU counts (**A**,**B**) changes in pH pH were monitored over a 4 days period. Initial CFU counts were ~9 logs.

Our results indeed demonstrated that UAD was a favorable medium for preserving *L. reuteri* cells. No drop in either CFU or pH was observed in a mixture of *L. reuteri* cells and UAD at 4 °C (Figure 5 A,B). By contrast a minimal drop was observed at 22 °C and some drop in CFU count was observed at 37 °C as well as a slight drop in pH from an initial pH of 7. A pH of 6.5 had already been found to be ideal for preserving cell viability (Figure 2C). Interestingly, comparable changes in CFU counts were observed with uMRS adjusted to pH 6.5 (Figure 5A,B). As expected, the MRS condition had significantly lower CFU drop on day 4 than almond drink and uMRS (*p* < 0.5) which indicated that the latter two conditions were more adept at preserving viability. However, MRS itself performed comparably well at 4 and 22 °C. The effects of pH on cell viability were found to be maximized at 37 °C and minimized at 4 and 22 °C, a result consistent with earlier studies demonstrating that temperature affects membrane proton permeability [25] and that growth temperature affects protein permeability at low temperatures [26].

## 4. Discussion

Adding 6% phosphate to MRS (MRS-KP) had been found to be sufficient for inducing autolysis in *L. reuteri* cells in prior studies [13,23]. In our prior study of the phosphate induction model we noticed a difference in protein expression patterns in the MRS-KP condition with respect to the MRS condition after 4 days of incubation at 37 °C [13]. Our hypothesis was that these differences in expression were induced by the excess phosphate and that expression changes could therefore be linked to the autolytic process. Our initial aim was thus to explore these differences in expression more thoroughly in order to identify novel cytoplasmic molecular determinants of autolysis in *L. reuteri* cells. Instead, we found that it was the untreated cells that underwent protein expression changes.

Based on our results we can conclude that pH is an important factor modulating phosphate induced autolysis, non-phosphate induced autolysis, and cell viability. Considering the results from uMRS, uMRS-KP, MRS, and MRS-KP, our hypothesis was that the loss in cell viability could be minimized by either increasing the buffer capacity to maintain the pH, a well-known solution for preserving strains of Lactobacilli [24], or by resuspending *L. reuteri* cells in a low nutrient environment that would prevent the production of lactic acid, thus maintaining a favorable pH for survival. This hypothesis is also consistent with data showing that depleting glucose from the media results in the preservation of a higher pH. This result was also consistent with our result that the high pH sugar free almond drink was a promising preservative for *L. reuteri*.

A crucial difference between the present study and earlier studies assessing almond drink [27,28], is that in the past studies, sugar was added in order to deliberately facilitate the fermentation of the almond drink. Importantly, that study group found a drop in viability (about 0.5 log drop to 7.5 log) after one day in storage at 4 °C [23,24,29]. Thus, simply adding the live cells to the almond drink appears to be a superior alternative to a fermented almond drink, since we were able to preserve 9 log cells over 4 days.

The original objective this study was to elucidate the downstream effects of autolysis inducing levels of phosphate on L. reuteri in a modest proteomics study of the cell cytoplasm. However, we have subsequently determined that the primary factor behind phosphate induced autolysis is simply pH, and that L. reuteri cells grown to the stationary phase are already “primed” for autolysis prior to the addition of phosphate and not as the result of the induction of a biochemical pathway by the phosphate itself. This “primed” state is obscured by a low final pH in a fermentation but is revealed by adjusting the pH to a more suitable neutral pH.

Phosphate induction of autolysis has been demonstrated in *Lactococcus* cells [11] and *Lactobacillus* species [9]. In the current study, we showed that the phosphate-induced model for *L. reuteri* must be updated to include pH as a factor. In addition, we demonstrated that the ideal pH for facilitating both phosphate induced and non-phosphate induced autolysis, pH 6.5, is also the ideal pH for preserving *L. reuteri* cells. Importantly, we have presented sugar-free alkaline food products as a possible medium for preserving *L. reuteri* survivability.

When reaching the stationary phase in the current model, *L. reuteri* cells are prepared for autolysis and have released autolysins into the media. However, the autolytic process is suppressed by a low pH that is generated by the lactic acid that has been produced. Low pH is detrimental to cells as they are no longer able to maintain homeostasis, and protons flood into the cell and inactivate cellular processes [26]. Low pH is thus lethal and leads to a low observed viability. The low pH also conformationally inactivates the autolysin molecules, which function best at a neutral pH. Hemocholinium-3 can also block autolysis in cells by interfering with the binding of the autolysins to lipoteichoic acid molecules, regardless of whether the autolysis is induced by phosphate or not. This is why a neutral pH leads to an increase in viability and also an increase in the level of both phosphate-induced autolysis and non-phosphate induced autolysis.

One of the limitations in the current work is that this study focuses on pH alone in the context of complex media environments. Additional work needs to be done in order to more fully reveal the various factors that could be influencing cell viability and autolysis. While the chemical components of almond drink have been determined to a large degree [30], more research should thus be carried out on the properties of almond drink in order to more fully understand which factors such as protein, fat, and fiber contents favor the preservation of *L. reuteri* cells.

Importantly, utilizing alkaline food matrices with low utilizable carbohydrates, such as almond drink, may be a good strategy for the design of a probiotic delivery system that enhances the survivability of L. reuteri. Further research would include the discovery of novel food matrices that fulfil these requirements.

## Data Availability

Not applicable.

## References

[1] Hill C., Guarner F., Reid G., Gibson G.R., Merenstein D.J., Pot B., Morelli L., Canani R.B., Flint H.J., Salminen S. (2014). The International Scientific Association for Probiotics and Prebiotics consensus statement on the scope and appropriate use of the term probiotic. Nat. Rev. Gastroenterol. Hepatol..

[2] Zheng J., Wittouck S., Salvetti E., Franz C.M.A.P., Harris H.M.B., Mattarelli P., O’Toole P.W., Pot B., Vandamme P., Walter J. (2020). A taxonomic note on the genus Lactobacillus: Description of 23 novel genera, emended description of the genus Lactobacillus Beijerinck 1901, and union of Lactobacillaceae and Leuconostocaceae. Int. J. Syst. Evol. Microbiol..

[3] Tomaro-Duchesneau C., Jones M.L., Shah D., Jain P., Saha S., Prakash S. (2014). Cholesterol assimilation by Lactobacillus probiotic bacteria: An in vitro investigation. Biomed. Res. Int..

[4] Lee J., Yang W., Hostetler A., Schultz N., Suckow M.A., Stewart K.L., Kim D.D., Kim H.S. (2016). Characterization of the anti-inflammatory Lactobacillus reuteri BM36301 and its probiotic benefits on aged mice. BMC Microbiol..

[5] Lakritz J.R., Poutahidis T., Levkovich T., Varian B.J., Ibrahim Y.M., Chatzigiagkos A., Mirabal S., Alm E.J., Erdman S.E. (2014). Beneficial bacteria stimulate host immune cells to counteract dietary and genetic predisposition to mammary cancer in mice. Int. J. Cancer.

[6] Zommiti M., Feuilloley M.G.J., Connil N. (2020). Update of Probiotics in Human World: A Nonstop Source of Benefactions till the End of Time. Microorganisms.

[7] Rice K.C., Bayles K.W. (2008). Molecular control of bacterial death and lysis. Microbiol. Mol. Biol. Rev..

[8] Ohmiya K., Sato Y. (1975). Promotion of Autolysis in Lactobacilli. Agric. Biol. Chem. Tokyo.

[9] Kang O.J., Vezinz L.P., Laberge S., Simard R.E. (1998). Some factors influencing the autolysis of Lactobacillus bulgaricus and Lactobacillus casei. J. Dairy Sci..

[10] Ramirez-Nunez J., Romero-Medrano R., Nevarez-Moorillon G.V., Gutierrez-Mendez N. (2011). Effect of Ph and Salt Gradient on the Autolysis of Lactococcus Lactis Strains. Braz. J. Microbiol..

[11] Lepeuple A.S., Van Gemert E., Chapot-Chartier M.P. (1998). Analysis of the bacteriolytic enzymes of the autolytic Lactococcus lactis subsp. cremoris strain AM2 by renaturing polyacrylamide gel electrophoresis: Identification of a prophage-encoded enzyme. Appl. Environ. Microbiol..

[12] Yamato M., Nakada R., Nakamura Y. (1998). Release of spirosin associated with potassium phosphate-induced autolysis in Lactobacillus reuteri DSM 20016. Microbiol. Res..

[13] Zimmerman T., Gyawali R., Ibrahim S. (2017). Autolyse the cell in order to save it? Inducing, then blocking, autolysis as a strategy for delaying cell death in the probiotic Lactobacillus reuteri. Biotechnol. Lett..

[14] Shukolyukov S.A. (2011). Native electrophoresis in cell proteomic: BN-PAGE and CN-PAGE. Cell Tissue Biol..

[15] Pan J.-Y., Wu H., Liu X., Li P.-P., Li H., Wang S.-Y., Peng X.-X. (2011). Complexome of Escherichia coli cytosolic proteins under normal native conditions. Mol. Biosyst..

[16] Li H., Pan J.-Y., Liu X.-J., Gao J.-X., Wu H.-K., Wang C., Peng X.-X. (2012). Alterations of protein complexes and pathways in genetic information flow and response to stimulus contribute to Escherichia coli resistance to balofloxacin. Mol. Biosyst..

[17] Gyawali R., Oyeniran A., Zimmerman T., Aljaloud S.O., Krastanov A., Ibrahim S.A. (2020). A comparative study of extraction techniques for maximum recovery of beta-galactosidase from the yogurt bacterium Lactobacillus delbrueckii ssp. bulgaricus. J. Dairy Res..

[18] Guan N., Liu L. (2019). Microbial response to acid stress: Mechanisms and applications. Appl. Microbiol. Biotechnol..

[19] Lund P., Tramonti A., De Biase D. (2014). Coping with low pH: Molecular strategies in neutralophilic bacteria. FEMS Microbiol. Rev..

[20] Hernández A., Larsson C.U., Sawicki R., van Niel E.W.J., Roos S., Håkansson S. (2019). Impact of the fermentation parameters pH and temperature on stress resilience of Lactobacillus reuteri DSM 17938. AMB Express.

[21] Verluyten J., Leroy F.D.R., de Vuyst L. (2004). Influence of Complex Nutrient Source on Growth of and Curvacin A Production by Sausage Isolate Lactobacillus curvatus LTH 1174. Appl. Environ. Microbiol..

[22] Riepe H.R., Pillidge C.J., Gopal P.K., McKay L.L. (1997). Characterization of the highly autolytic Lactococcus lactis subsp. cremoris strains CO and 2250. Appl. Environ. Microbiol..

[23] Bernat N., Chafer M., Chiralt A., Gonzalez-Martinez C. (2015). Probiotic fermented almond “milk” as an alternative to cow-milk yoghurt. Int. J. Food Stud..

[24] Ter Kuile B.H., Wiemer E.A.C., Michels P.A.M., Opperdoes F.R. (1992). The electrochemical proton gradient in the bloodstream form of Trypanosoma brucei is dependent on the temperature. Mol. Biochem. Parasitol..

[25] Lee J., Townsend J.A., Thompson T., Garitty T., De A., Yu Q., Peters B.M., Wen Z.T. (2018). Analysis of the Cariogenic Potential of Various Almond Milk Beverages using a Streptococcus mutans Biofilm Model in vitro. Caries Res..

[26] Bernat N., Chafer M., Chiralt A., Gonzalez-Martinez C. (2015). Development of a non-dairy probiotic fermented product based on almond milk and inulin. Food Sci. Technol. Int..

[27] van de Vossenberg J.L.C.M., Driessen A.J.M., da Costa M.S., Konings W.N. (1999). Homeostasis of the membrane proton permeability in Bacillus subtilis grown at different temperatures. Biochim. Biophys. Acta (BBA) Biomembr..

[28] Hutkins R.W., Nannen N.L. (1993). Ph Homeostasis in Lactic-Acid Bacteria. J. Dairy Sci..

[29] Lipan L., Rusu B., Sendra E., Hernández F., Vázquez-Araújo L., Vodnar D.C., Carbonell-Barrachina Á.A. (2020). Spray drying and storage of probiotic-enriched almond milk: Probiotic survival and physicochemical properties. J. Sci. Food Agric..

[30] Adams M. (1959). Bacteriophages.

